# Our Animal Friends

**Published:** 1891-12

**Authors:** 


### BOOK NOTICES.

Our Animal Friends. A monthly journal published by the American Society for the Prevention of Cruelty to Animals. Headquarters, 100 East 22d Street, New York, John P. Haines, President. Subscription, $1.00 a year, strictly in advance.

The September number of this useful publication has been sent us. It contains a fine portrait of Henry Bergh, the famous champion of animals. There are twenty-three pages of instructive reading-matter, referring to dumb animals and inculcating kind treatment of them. This Journal should be in every amily where there are children to instruct them in ways of kindness and merciful treatment of animals.

